# Understanding caregiver preferences for firearm locking devices in a pediatric emergency department

**DOI:** 10.1186/s40621-025-00568-y

**Published:** 2025-02-28

**Authors:** Tyler Lennon, Samaa Kemal, Sanjana Shankar, Robert Tunick, Doug Lorenz, Jennifer A. Hoffmann

**Affiliations:** 1 Division of Pediatric Emergency Medicine, Department of Pediatrics, Ann & Robert H. Lurie Children’S Hospital of Chicago, Chicago, IL USA; 2 Division of Pediatric Emergency Medicine, Department of Pediatrics, Medical College of Wisconsin, 999 N, 92nd Street, Milwaukee, WI 53226 USA; 3 Department of Bioinformatics and Biostatistics, University of Louisville, Louisville, KY USA

**Keywords:** Firearms, Mental health, Safe storage, Injury prevention, Pediatrics

## Abstract

**Background:**

Around 40% of US households with children have a firearm kept in the home. This study sought to describe firearm storage practices and locking device preferences among caregivers of children presenting to a pediatric emergency department (ED).

**Methods:**

We conducted a cross-sectional survey of caregivers of children presenting to a pediatric ED who endorsed having a firearm in the home from August 2023 to May 2024. A self-administered electronic survey inquired about current firearm storage practices and locking device preferences. Caregivers who endorsed any unsafe firearm storage practice (i.e., firearm stored unlocked, loaded, and/or with ammunition) were offered, based on their preference, a free cable gun lock, lock box, or gun safe along with safe firearm storage education materials. Caregivers given a device were surveyed 30 days later to reevaluate firearm storage behavior. McNemar’s test was used to evaluate differences in reported baseline locking device use. Wilcoxon signed rank test was used to evaluate changes in storage behaviors (locked, unloaded, and stored separate from ammunition) from baseline to 30-day follow-up.

**Results:**

Of 139 caregivers with a firearm in the home, 91% (*n* = 126) reported having a handgun and 41% (*n* = 57) reported storing firearms with triple safe storage. Safes/vaults were more frequently used (40%, *n* = 56) than cable gun locks (20%, *n* = 28) (*p* = 0.003). Factors involved in caregiver preference for firearm locking devices were: speed of access to firearms (47%, *n* = 66), strength of the device (45%, *n* = 63), and cost of device (42%, *n* = 58). Seventeen caregivers were provided a free device based on preference: 15 gun safes, 2 lock boxes, and 0 cable gun locks. At 30-day follow up, 70.6% (*n* = 12) of eligible caregivers responded, and the proportion of respondents who reported storing all firearms locked increased significantly from baseline (from 67 to 100%, *p* = 0.036).

**Conclusions:**

Caregivers in a pediatric ED reported using safes most frequently and, when offered a device, preferred safes over other locking devices. After being provided a free device of their preference, all caregivers who completed follow-up surveys reported all firearms were locked. Healthcare and community organizations should align resources with caregiver preferences.

**Supplementary Information:**

The online version contains supplementary material available at 10.1186/s40621-025-00568-y.

## Introduction

Gun violence is the number one cause of death for children as of 2020 [1]. Approximately 40% of US households with children have a firearm kept in the home [2]. Safe storage of firearms has been shown to decrease unintentional firearm injury as well as adolescent suicide [3, 4]. The American Academy of Pediatrics recommends firearms be stored locked, unloaded, and stored separate from ammunition [5]. However, currently an estimated 4.6 million children live in households with at least one unsafely stored firearm [2].

A variety of locking devices are available to firearm owners, including cable locks, lock boxes, and gun safes. Lower cost devices, such as cable locks where a cable goes through the weapon and prevents it from being loaded and fired, have often been selected for public health interventions to maximize distribution in the setting of limited resources. For example, cable locks have been distributed at no cost to families in health care settings, police departments, and community centers [6]. However, focus groups and surveys have revealed that these devices are often considered a nuisance by firearm-owning parents and are rarely used [7]. In contrast, most firearm owners would prefer and at least consider using a lock box given quicker access to the firearm in an emergency compared to a cable lock [8]. Furthermore, a recent systematic review found that gun safes were the device most frequently used by US firearm owners given their strength and speed of access to the firearm [9]. Currently, it is unknown what locking devices are used by caregivers of children seen in an emergency department (ED) setting who report a firearm in the home, as well as what locking devices caregivers, especially those with unlocked firearms in the home, would be open to using. Previous research in the pediatric ED has had success by focusing on lethal means counseling often paired with device distribution [10, 11], however no studies have evaluated caregivers preferred locking devices which may increase safe storage uptake. Furthermore, the ED setting is a particularly important setting given this is where patients present in a mental health crisis or with an unintentional injury and therefore provides an important opportunity to counsel families and ensure safety. An understanding of firearm storage practices and preferences for locking devices will inform interventions in the pediatric ED.

Thus, we aimed to understand firearm storage practices and preferences for locking devices among caregivers in an urban pediatric ED. Among caregivers who endorsed unsafe firearm storage practices at baseline, we also aimed to assess changes in firearm storage practices 30 days after provision of a preferred locking device and safety education in the ED.

## Methods

### Study design, setting, and participants

We conducted a cross-sectional survey of parents and caregivers of children seen in the ED of an urban academic children’s hospital from August 2023 to May 2024 who reported that a firearm was kept in their home. Eligible participants were at least 18 years old, a parent or caregiver of a child who was being cared for in the ED, and endorsed that at least one firearm was kept in their home. The study was reviewed and approved by the lead author’s hospital Institutional Review Board.

## Study procedure and measures

A convenience sample of caregivers was selected. Flyers in English and Spanish advertising the survey were posted in each patient care room in the ED (Appendix A). Caregivers opted-in to survey participation by using their personal device to scan a quick-response (QR) code on the flyer and no prompting by team members or research staff was used. Surveys were hosted using REDCap, an online survey platform [12, 13]. Survey content included: the number and types of firearms in the home; current storage practices including what devices, if any, were currently used; firearm locking device preferences; and reasons for those preferences (Appendix B). Triple safe storage was defined as firearms stored locked, unloaded, and separate from ammunition [5].

Caregivers who indicated at least one firearm was stored unsafely (i.e., unlocked, loaded, and/or stored with ammunition) were offered a free locking device of their choosing: a cable lock, lock box, or gun safe. During business hours, an automated message was sent to research staff to provide the device of their choosing. Outside of business hours, caregivers were directed to ask the treating ED clinician or nurse for the device of their choosing. These caregivers were given educational materials on safe firearm storage as well as manufacturer user instructions for the selected device. Caregivers provided a device were sent a 30-day follow-up survey, by text message or email, to assess firearm storage practices and device use (Appendix C). Caregivers were compensated $5 for completion of the initial survey and $10 for completion of the follow-up survey.

### Statistical analysis

We used descriptive statistics to summarize caregiver firearm storage behaviors and preferences. We used McNemar’s test to evaluate differences in reported baseline locking device use. Among caregivers who reported unsafe storage practices and received a free locking device, we used Wilcoxon signed rank test to evaluate changes in individual storage behaviors (locked, unloaded, and stored separate from ammunition) and McNemar’s test to evaluate changes in the proportion of caregivers who reported triple safe storage practices at baseline and 30-day follow-up. Analyses were conducted in the open-source R software environment (v 4.4.0) [14].

## Results

Of 139 caregivers who endorsed that a firearm was kept in the home, 47% (*n* = 65) reported one firearm and 91% (*n* = 126) reported a handgun (Table 1). Triple safe storage was reported by 41% (*n* = 57) of caregivers. Most caregivers reported storing firearms locked (81%, *n* = 113), with less reporting storing firearms unloaded (65%, *n* = 91) or separate from ammunition (53%, *n* = 74). Regarding locking devices currently in use, 40% (*n* = 56) of caregivers reported using a safe/vault, while 20% (*n* = 28) of caregivers reported using a cable gun lock (*p* = 0.003) (Table 2). Factors involved in caregiver preferences for locking devices were speed of access to the firearm (47%, *n* = 66), strength of the device (45%, *n* = 63) and cost of the device (42%, *n* = 58) (Table 1).

**Table 1:** Firearm storage practices and preferences among caregivers in the pediatric emergency department with a firearm in the home

	*N* (%) *N* = 139
Type of firearm in the home	
Handgun	126 (91)
Long gun	29 (21)
Other	5 (4)
Number of firearms in the home	
One	65 (47)
Two	24 (17)
Three	13 (9)
Four or more	17 (12)
Missing	20 (14)
Storage practices	
Triple safe storage for all firearms	
Yes	57 (41)
No	82 (59)
Firearms stored locked	
All	113 (81)
Some	11 (8)
None	8 (6)
Missing	7 (5)
Firearms stored unloaded	
All	91 (65)
Some	20 (14)
None	21 (15)
Missing	7 (5)
Firearms stored separate from ammunition	
All	74 (53)
Some	25 (18)
None	33 (24)
Missing	7 (5)
Factor(s) involved in device preference^1^	
Speed of access to firearm	66 (47)
Strength of device	63 (45)
Cost of device	58 (42)
Ease of obtaining device	50 (36)
Ease of access to firearm	47 (34)
Compatibility with firearms	42 (30)
Size of device	41 (29)
Portability of device	27 (19)
Appearance of device	14 (10)

^1^ Percentages add to over 100% because caregivers could select more than one factor involved in device preference

**Table 2:** Current use of and preferences for firearm locking devices among caregivers in the pediatric emergency department

Firearmlocking device	Device currently being used, *N* (%) *N* = 139	Device caregiver would consider using, *N* (%) *N* = 139	Most preferred device if provided for free, *N* (%) *N* = 139
Cable gun lock	28 (20)	43 (31)	24 (17)
In-vehicle lock	6 (4)	42 (30)	25 (18)
Trigger lock	17 (12)	43 (31)	23 (17)
Lock box	40 (29)	64 (46)	38 (27)
Gun safe or vault	56 (40)	77 (55)	73 (53)
Gun cabinet	12 (9)	40 (29)	29 (21)
No device	12 (9)	11 (8)	5 (4)

Of 82 caregivers who were eligible to receive a free locking device due to one or more unsafe storage behaviors, 33 requested a device within the survey, with 69.7% (*n* = 23) of these caregivers requesting a gun safe and 30.3% (*n* = 10) requesting a lock box. No caregivers requested a cable gun lock. There were 17 free devices distributed, with 16 provided by research staff and 1 provided by an ED clinician. The distributed devices included 15 gun safes and 2 lock boxes.

After the 30-day follow-up period, 70.6% (*n* = 12) of eligible caregivers who received a free device completed the follow-up survey. The proportion of caregivers who reported all firearms were locked significantly increased from baseline (from 67 to 100%, *p* = 0.036). There were no significant differences between baseline and 30-day follow-up in the proportion of caregivers who reported that firearms were stored unloaded (33% vs. 25%, *p* = 0.62), that firearms were stored separate from ammunition (25% vs. 33%, *p* = 0.62), or that firearms were stored using triple safe storage practices (0% vs. 17%, *p* = 0.48).

## Discussion

In this survey of caregivers in an urban pediatric ED who report a firearm in the home, most reported a handgun and less than half reported storing firearms using triple safe storage practices. Caregivers most frequently used safes/vaults to store firearms, and, of those who requested a device, most requested a gun safe. Speed of access to the firearm was the most frequent factor caregivers considered when choosing a locking device. Among caregivers who endorsed unsafe storage practices who were provided with a free locking device of their choice and safe storage educational materials, 100% of those who completed follow-up surveys reported storing all firearms locked at 30-day follow-up.

Findings from this study were similar to a recent national study that showed only 44.1% of firearm owners with children store firearms in the safest manner (locked and unloaded) [2] and that speed of access and convenience are more important to firearm-owning caregivers than safety when choosing a locking device [15, 16]. Additionally, findings from this study were similar to previous studies that showed improvement in safe storage behavior after free locking device provision and firearm safety counseling [17, 18]. Uniquely, after the 30-day follow-up, all caregivers in this study reported storing all firearms locked, however, there was no significant change in reported triple safe storage behavior. While triple safe storage is optimal for safety, many caregivers have reasons for not engaging in triple safe storage such as speed of access [15, 16]. However, an improvement in firearm storage from unlocked to locked is still a meaningful improvement in safety.

Our findings have several important implications. Free cable gun locks have often been distributed in health care and community settings [6], given their low cost and portability, however, our findings suggest that these devices do not align with the preferences of caregivers who report a firearm in the home. Mechanisms to broaden access to preferred devices such as gun safes/vaults may increase uptake of device use among caregivers who report a firearm in the home. For example, a national survey of firearm owners found that respondents desired coupons for at least 25% off a locking device [15]. Our findings support the need for advocacy and injury prevention efforts to increase distribution of preferred locking devices in healthcare settings such as the pediatric ED. Firearm death by suicide and unintentional injury have both been decreased with improvement in safe storage uptake [3, 4] and the pediatric ED provides a unique setting to provide these devices and counseling as patients often present in a mental health crisis or with unintentional injury. Additionally, studies with larger sample sizes are needed to determine whether provision of preferred locking devices, relative to cable gun locks alone, increases uptake of triple safe storage practices among caregivers who report a firearm in the home. As several studies have demonstrated suboptimal uptake of triple safe storage practices with counseling and device distribution alone [19–21], future research should also investigate novel approaches, such as digital decision aids [22] during the ED visit and sending reminders or “nudges” to caregivers following the ED visit [6].

This study has important limitations. There is the potential for selection bias given that caregivers self-selected into the study. Caregivers who completed surveys may have been more interested and engaged in the topic of safe storage and those with potential law enforcement or child protection service involvement may have been less likely to opt into the survey. In addition, there is potential for social desirability bias which could have led to over-reporting of safe storage practices. We only offered the survey in English and Spanish, so results may not generalize to caregivers with other language preferences. To maintain anonymity, given the sensitive nature of the topic, we did not collect demographic characteristics of caregivers or link survey data to electronic health record data. Additionally, 33 caregivers requested a free locking device, while only 17 devices were provided. This was mainly due to device requests outside of research staff hours, but other reasons such as the caregiver listing the wrong room number or being discharged prior to delivering the device were also relevant. Caregivers were instructed to ask their ED care provider to provide the device, but this happened on only one occasion. This highlights that caregivers may be hesitant to bring up their desire for a device with their ED clinician, whereas automated notifications may have been more successful. Finally, this study was conducted in a single urban pediatric ED and may not be generalizable to other populations or settings, particularly where firearms other than handguns, such as long guns, are more prevalent.

## Conclusions

In an urban pediatric ED, fewer than half of caregivers who reported a firearm in the home reported triple safe storage practices. Caregivers preferred higher quality devices such as gun safes and lock boxes compared to cable locks. After being provided a free device of their preference, 100% of caregivers who completed follow-up surveys reported storing all firearms locked at 30-day follow-up. Healthcare and community organizations should strive to align resources with the preferences of caregivers who report a firearm in the home to enhance uptake of safe firearm storage practices.

## Electronic supplementary material

Supplementary Material 1

Supplementary Material 2

## Data Availability

The datasets used and/or analyzed during the current study are available from the corresponding author on reasonable request.
