# Supplementary material for: Understanding caregiver preferences for firearm locking devices in a pediatric emergency department
Source: Inj Epidemiol. 2025 Feb 28;12:13. doi: 10.1186/s40621-025-00568-y (PMC11869430; doi:10.1186/s40621-025-00568-y)
Supplement: Supplementary file 1 — Supplementary Material 1 [file 40621_2025_568_MOESM1_ESM.docx]

**Appendix B: Firearm Storage Initial Survey**

Thank you for being willing to share your perspective with us. The goal of this survey is to understand parent and caregiver preferences around locking devices for secure storage of personal firearms. This information will be used to think about how to best prevent firearm-related injuries in children.

Your decision to complete this survey is completely voluntary. By completing it, you are

consenting to participate and agreeing for your responses to be used for research

purposes. In order to participate you must be proficient in English or Spanish. Your answers will be kept confidential.

1. Have you taken this survey before?
   - Yes
   - No

*If the participant answers yes, the survey will end as they are not eligible.*

1. Are you a parent or caregiver of a child?
   - Yes
   - No

1. Are you 18 years of age or older?
   - Yes
   - No

1. Is there a gun kept in your home?
   - Yes
   - No

*If the participant answers Yes to both questions, they will continue through the survey. If the participant answers No to either question, the survey will end as they are not eligible.*

The following questions will ask about the firearms currently in your home.

1. How many firearms are in your home? (Number box - Integer)

1. What type(s) of firearm(s) are in your home? Check all that apply.
   1. Handguns (including revolvers and pistols)
   2. Long guns
   3. Other (free text)

The following questions will ask about how firearms are currently stored in your home.

1. Are firearm(s) stored locked?
   - All firearms are stored locked
   - Some firearms are stored locked
   - No firearms are stored locked

1. Are firearm(s) stored unloaded?
   - All firearms are stored unloaded
   - Some firearms are stored unloaded
   - No firearms are stored unloaded

1. Are firearm(s) stored separate from your ammunition?
   - All firearms are stored separate from ammunition
   - Some firearms are stored separate from ammunition
   - No firearms are stored separate from ammunition

Many organizations and community programs are interested in giving out firearm locking

devices for free or at a reduced cost – but they aren’t sure which types of devices firearm

owners prefer. When we say “firearm locking devices” we are referring to storage and/or staging devices that limit unauthorized access to firearms. The following questions will ask about your opinions and preferences for firearm locking devices.

1. What kind of firearm locking device(s) are you currently using?
2. Cable gun lock
3. In-vehicle lock
4. Trigger lock
5. Lock box
6. Gun safe or vault
7. Gun cabinet
8. None

1. *(If endorses use of any firearm locking device in preceding question)* Does your [insert name of firearm locking device] have any of the following features? *(Repeat for each answer selected to question 10)*
2. Key
3. Number PIN
4. Dial
5. Biometric/fingerprint

1. Which of the following firearm locking devices would you be open to using?
2. Cable gun lock
3. In-vehicle lock
4. Trigger lock
5. Lock box
6. Gun safe or vault
7. Gun cabinet
8. None

1. Which of the following firearm locking devices would you most prefer to use if the device were provided to you free of charge?
   - Cable gun lock
   - In-vehicle lock
   - Trigger lock
   - Lock box
   - Gun safe or vault
   - Gun cabinet
   - None

1. What factors are involved in your preference for firearm locking devices? Check all that apply.
   1. Cost of device
   2. Ease of obtaining device
   3. Speed of access to firearm
   4. Ease of access to firearm
   5. Size of device
   6. Compatibility with firearms
   7. Portability of device
   8. Strength of device
   9. Appearance of device
   10. Other (free text)

1. Would you like to tell us anything else about firearm locking devices? (Free Text)

End of survey

Thank you for taking part in our survey. You are eligible to receive a $5 electronic gift card for your participation in this study. Please enter your e-mail address here if you would like to receive this. Your e-mail address will not be linked to your survey results.

*If participant endorsed any unsafe firearm storage above (ANY firearms unlocked, loaded or stored with ammunition), they will be directed to this page:*

Thank you for taking part in our survey. You are eligible to receive a free firearm locking device today. We have cable gun locks, lock boxes and gun safes available in the emergency department and can be distributed to you during today’s visit. Would you like one of our free firearm locking devices? (Yes/No)

*If yes, they will be linked to a new page.* Please choose one of the following firearm locking devices: cable gun lock, lock box, or gun safe. Please let one of your providers know which device you chose so they can provide this to you before you leave the Emergency Department today.

We would like to understand if you are using the device given to you today. Can we contact you with a brief follow-up survey in 1 month? You will be eligible for a $10 gift card for completing the follow-up survey. Please provide your phone number to receive a text message link in one month to the follow-up survey (you will not be called by the study team) and/or provide your email address to receive the follow-up survey in one month by email. (Yes/No). *If yes, provide two different fields to enter information for email or phone number. If no, proceed to below.*

*Education Statement for End of Survey:*

The American Academy of Pediatrics recommends that all firearms in households with children should be unloaded and in locked storage, with ammunition stored separately. If you would like more information about safe firearm storage, please ask your provider for our flyer.

**Appendix C:** **Firearm Storage Follow-up Survey**

Thank you for being willing to share your perspective with us. The goal of this survey is to understand if you have been using the free firearm locking device given to you one month ago during your emergency department visit.

Your decision to complete this survey is completely voluntary. By completing it, you are

consenting to participate and agreeing for your responses to be used for research

purposes. In order to participate you must be proficient in English or Spanish. Your answers will be kept confidential.

1. Which of the following firearm locking devices did you receive from the Lurie Children’s emergency department approximately one month ago?

- Cable gun lock
- Lock box
- Gun safe
- None

*If participant chooses none, the survey will end.*

1. Are you using the firearm locking device that was provided to you at that visit?

- Yes
- No

1. *(If participant answers No to question 2)* Please share the reason(s) why you are not using the firearm locking device provided to you?
2. It is difficult to access to my firearm
3. It takes too long to access my firearm
4. The device is not compatible with my firearm
5. The device is too big
6. The device is too small
7. I am storing my firearm using another device
8. Other (free text)

1. What other firearm locking device(s), if any, are you currently using?
2. Cable gun lock
3. In-vehicle lock
4. Trigger lock
5. Lock box
6. Gun safe or vault
7. Gun cabinet
8. None

1. Are firearm(s) stored locked?

- All firearms are stored locked
- Some firearms are stored locked
- No firearms are stored locked

1. Are firearm(s) stored unloaded?

- All firearms are stored unloaded
- Some firearms are stored unloaded
- No firearms are stored unloaded

1. Are firearm(s) stored separate from your ammunition?

- All firearms are stored separate from ammunition
- Some firearms are stored separate from ammunition
- No firearms are stored separate from ammunition

1. Is there any other feedback you would like to provide on the firearm storage device provided to you at your last visit? (Free text)

Thank you for taking part in our survey. You are eligible to receive a $10 electronic gift card for your participation in this study. Please enter your e-mail address here if you would like to receive this. Your e-mail address will not be linked to your survey results.
