# Supplementary material for: Understanding caregiver preferences for firearm locking devices in a pediatric emergency department
Source: Inj Epidemiol. 2025 Feb 28;12:13. doi: 10.1186/s40621-025-00568-y (PMC11869430; doi:10.1186/s40621-025-00568-y)
Supplement: Supplementary file 2 — Supplementary Material 2 [file 40621_2025_568_MOESM2_ESM.pdf]

Have a firearm in your home?

You are invited to participate in an ANONYMOUS study to help us better understand how firearms are stored in the homes of children and what firearm storage methods families prefer.

STUDY ELIGIBILITY

- You are a parent or caregiver of a child
- You are 18 years of age or older
- You have a firearm in your home

STUDY PROCESS

- Voluntary participation by taking a survey
- All surveys are anonymous and will not be linked to your ER visit
- The survey will take 5-10 minutes
- Scan the QR code below to take the survey on your phone or tablet

$5 electronic gift card will be given for participation

Ann & Robert H. Lurie
Children's Hospital of Chicago®

This study is Ann & Robert H. Lurie Children's Hospital IRB # 2023-6024, Understanding Firearm Storage Practices and Preferences in the Pediatric Emergency Department, Samaa Kemal and Tyler Lennon. The content of this flyer has been approved by Ann & Robert H. Lurie Children's Hospital IRB.
